# An update on the long-term outcomes of prenatal dexamethasone treatment in congenital adrenal hyperplasia

**DOI:** 10.1530/EC-22-0400

**Published:** 2023-03-15

**Authors:** Annelies van’t Westeinde, Leif Karlsson, Valeria Messina, Lena Wallensteen, Manuela Brösamle, Giorgio Dal Maso, Alessandro Lazzerini, Jette Kristensen, Diana Kwast, Lea Tschaidse, Matthias K Auer, Hanna F Nowotny, Luca Persani, Nicole Reisch, Svetlana Lajic

**Affiliations:** 1 Department of Women’s and Children’s Health, Karolinska Institutet and Division of Pediatrics, Unit for Pediatric Endocrinology and Metabolic Disorders, Karolinska University Hospital, Stockholm, Sweden; 2 European Patient Advocacy Group for Adrenal Diseases, European Reference Network on Rare Endocrine Conditions (Endo ERN), Endo ERN Coordinating Centre, Leiden, The Netherlands; 3 ArfSAG (Associazione Refionale Famiglie Sindrome Adreno Genitale) c/o Unita Operativa di Pediatria, Azienda Ospedaliero Universitaria di Bologna, Policlinico S Orsala-Malpighi, Bologna, Italy; 4 Spanish Association of Congenital Adrenal Hyperplasia (CAH), Spain; 5 ePAG & Chair of Danish Addison Patient Association, Aarhus, Denmark; 6 Dutch Adrenal Society NVACP, Nijkerk, The Netherlands; 7 Department of Endocrinology, Medizinische Klinik IV, Klinikum der Universität München, Munich, Germany; 8 Department of Medical Biotechnology and Translational Medicine, University of Milan, Milan, Italy; 9 Department of Endocrine and Metabolic Diseases, Istituto Auxologico Italiano IRCCS, Milan, Italy

**Keywords:** CAH, dexamethasone, brain development, first trimester, prenatal treatment, treatment safety

## Abstract

First-trimester prenatal treatment with glucocorticoid (GC) dexamethasone (DEX) in pregnancies at risk for classic congenital adrenal hyperplasia (CAH) is associated with ethical dilemmas. Though effective in reducing virilisation in girls with CAH, it entails exposure to high doses of GC in fetuses that do not benefit from the treatment. The current paper provides an update on the literature on outcomes of prenatal DEX treatment in CAH cases and unaffected subjects. Long-term follow-up research is still needed to determine treatment safety. In addition, advances in early prenatal diagnostics for CAH and sex-typing as well as studies assessing dosing effects of DEX may avoid unnecessary treatment and improve treatment safety.

## Introduction

Congenital adrenal hyperplasia (CAH) due to 21-hydroxylase (21-OH) deficiency occurs in around 1:10,000–1:15,000 newborns (1, 2, 3, 4, 5). It is caused by mutations in the *CYP21A2* gene coding for the 21-OH enzyme, which is required for the conversion of cholesterol to cortisol and aldosterone, rendering it either partly or completely ineffective (2, 3). Patients with classic CAH, therefore, experience glucocorticoid (GC) and mineralocorticoid deficiency already prenatally and prompt treatment with cortisol and aldosterone is needed to prevent salt-wasting (SW) crises and neonatal death in the most severe cases. Although the disease is manageable with life-long medication, optimal replacement is challenging and periods of supra- or infra-physiological levels of cortisol may frequently occur during any period in the patient’s life. The extent of the deficiency depends on the genetic variant causing the malfunctioning of the 21-OH enzyme, in which the mildest allele determines the severity of the disease, ranging from mild non-classic (NC), to a null-genotype with complete loss of function (6). NC CAH may only be detected later in life, while the more severe simple-virilising (SV) and SW CAH are detected either through a neonatal screening programme, as a result of experienced adrenal crises during the neonatal period, or virilisation in females (4, 7).

Already *in utero*, the lack of cortisol causes a reduction, or complete absence of negative feedback on the hypothalamus–pituitary–adrenal (HPA) axis, resulting in the over-production of adrenocorticotropic hormone (ACTH). The excess ACTH is shunted towards the androgen production pathways in the adrenal cortex and thus leads to an overproduction of dehydroepiandrosterone and other adrenal androgens (8, 9, 10). During fetal life, the external genitalia start developing around gestational week (GW) 7, and in females, suppression of adrenal androgens by cortisol is required to ensure female sex development and prevent genital virilisation. In other words, excessively high androgen levels will cause the female genitalia to develop towards the male phenotype. The lack of HPA axis inhibition can, therefore, result in severe virilisation, including enlarged clitoris and labial fusion, to the extent that a girl with CAH is sometimes assigned the wrong sex at birth. The extent of virilisation depends on the CAH genotype and is classified according to Prader stages. Virilised genitalia may cause psychological and physiological problems for the patients (11, 12).

To ameliorate virilisation, genital surgery may be performed either at toddler age, around 1.5 years, or during puberty (13, 14, 15). Briefly, outcomes of early surgical interventions are sub-optimal and in addition entail a procedure for a non-life-threatening condition performed without the patient being able to give consent. Though short-term surgical complications may be fixed (13, 16, 17), long-term negative effects related to sexual function may not be avoided and are frequently reported (15), even if surgery is performed at a later age (18, 19, 20). Alternatively, patients and parents may opt out of surgery. There is some indication that GC treatment alone is able to reduce clitoris length to less than half the size (21), questioning the necessity of early surgery. It is important to provide education and psychological support to parents and un-operated girls during their upbringing. Future studies should investigate the girls’ experiences, quality of life and psychological outcomes of opting in or out of early surgery.

For couples at risk of having a child with SV or SW CAH, the synthetic GC dexamethasone (DEX) may be given to the pregnant woman to prevent/reduce virilisation in a girl with CAH. This treatment has been offered since the 1980s (22). However, prenatal exposure to DEX also entails risks. Worldwide, only a few centres have conducted long-term follow-up studies regarding the outcome of prenatal DEX treatment in the context of at-risk pregnancies (23, 24, 25, 26, 27). Previous publications from the Swedish group have reviewed the results of these studies (28, 29, 30). The present paper provides an update with the latest results on prenatal DEX outcomes, placed in the context of prenatal development and in particular brain development.

## Prenatal dexamethasone treatment in CAH

**Figure 1:**
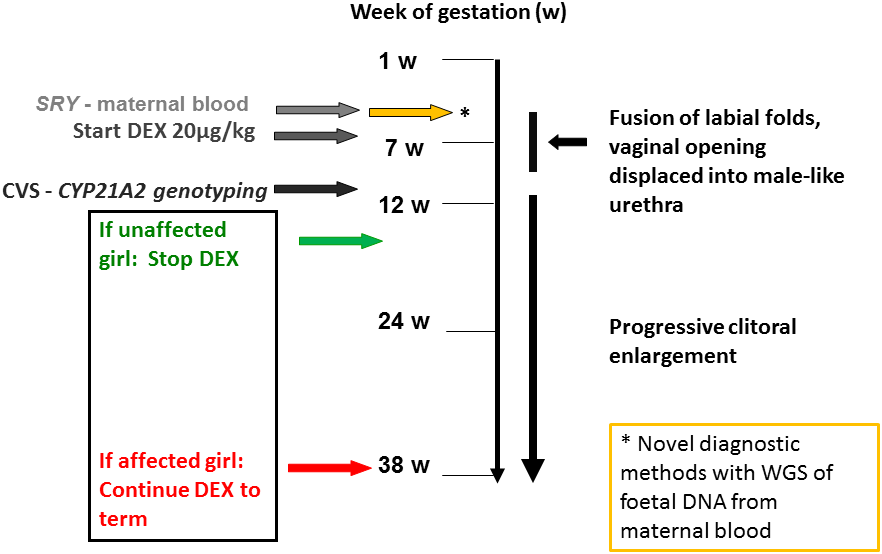
Overview of the prenatal dexamethasone treatment in pregnancies at risk for CAH. SRY analysis from maternal blood determines the sex of the fetus prior to DEX treatment. Treatment is only started if the fetus is a girl. Novel tests using whole genome sequencing (WGS) of fetal DNA obtained from maternal blood may in the future limit treatment to girls with CAH (yellow arrow). Currently, genotyping for *CYP21A2* from chorionic villus sampling (CVS) is done at week 12, which determines the CAH diagnosis. DEX treatment is stopped in the case of an unaffected girl and continued until term in the case of a girl with CAH. If untreated, genitalia develop towards the male phenotype, as indicated on the right side of the figure.

DEX needs to be administered during the first trimester and started before GW7 in order to be effective in preventing the closure of the labioscrotal folds and other processes leading to a male sex phenotype (Fig. 1). Because fetal sex-typing from cell-free DNA derived from maternal blood is possible before treatment needs to be initiated, the male fetuses can be spared from DEX exposure (13). However, this technique has only recently been adapted to clinical settings, and as a result, a large cohort of subjects worldwide includes first-trimester treated boys with CAH as well as children without CAH.

After GW12, genotyping for CAH is possible, and treatment is only continued until term in the case of girls with CAH. Unlike maternal cortisol, DEX is not metabolized by the enzyme HSD11B type 2 and therefore able to cross the placenta to enter the fetal bloodstream. It has a long half-life and binds exclusively to the glucocorticoid receptor (GR), making it a potent agent to regulate the HPA axis prenatally and suppress adrenal androgen excess. It is unclear which exact dose is required to sufficiently suppress adrenal androgens in girls with CAH, but currently, a standard dose of 20 µg/kg/day (max 1.5 mg/day) is given (22) to the pregnant woman. The effect of this dose has not been systematically evaluated but may result in substantially higher cortisol levels than normally present in healthy fetuses (32, 33), and recently, it was suggested that the dose may be three times higher than needed (32). Moreover, some debate has arisen regarding the required timing of the DEX exposure. One retrospective study, based on four cases, proposed that the timing of exposure could potentially be limited to the window of partitioning or the time of urogenital cleavage, instead of being given during the entire pregnancy (34). However, this does not prevent clitoromegaly and the child may later still require surgery. Although DEX is widely available worldwide, the occurrence of treatment varies substantially between countries (35).

The major concern of first-trimester DEX treatment is that it needs to be started before genotyping for CAH is possible (31, 36, 37). Thus, healthy girls and boys may be exposed to unnecessarily high GC levels during this sensitive developmental period. Prenatal treatment in CAH is therefore controversial and currently only recommended in research settings where participants can be followed up closely (5). In Sweden participants have been followed since 1999 in the PREDEX study (38), and in France there is an ongoing national multicenter study (31). Careful evaluation of the outcomes of both healthy first-trimester treated children, first-trimester treated boys with CAH, full-term treated girls with CAH and untreated CAH children is required to determine treatment safety and weigh the risks and benefits.

## The manifold role of cortisol: points of interference and clinically relevant endpoints

**Figure 2:**
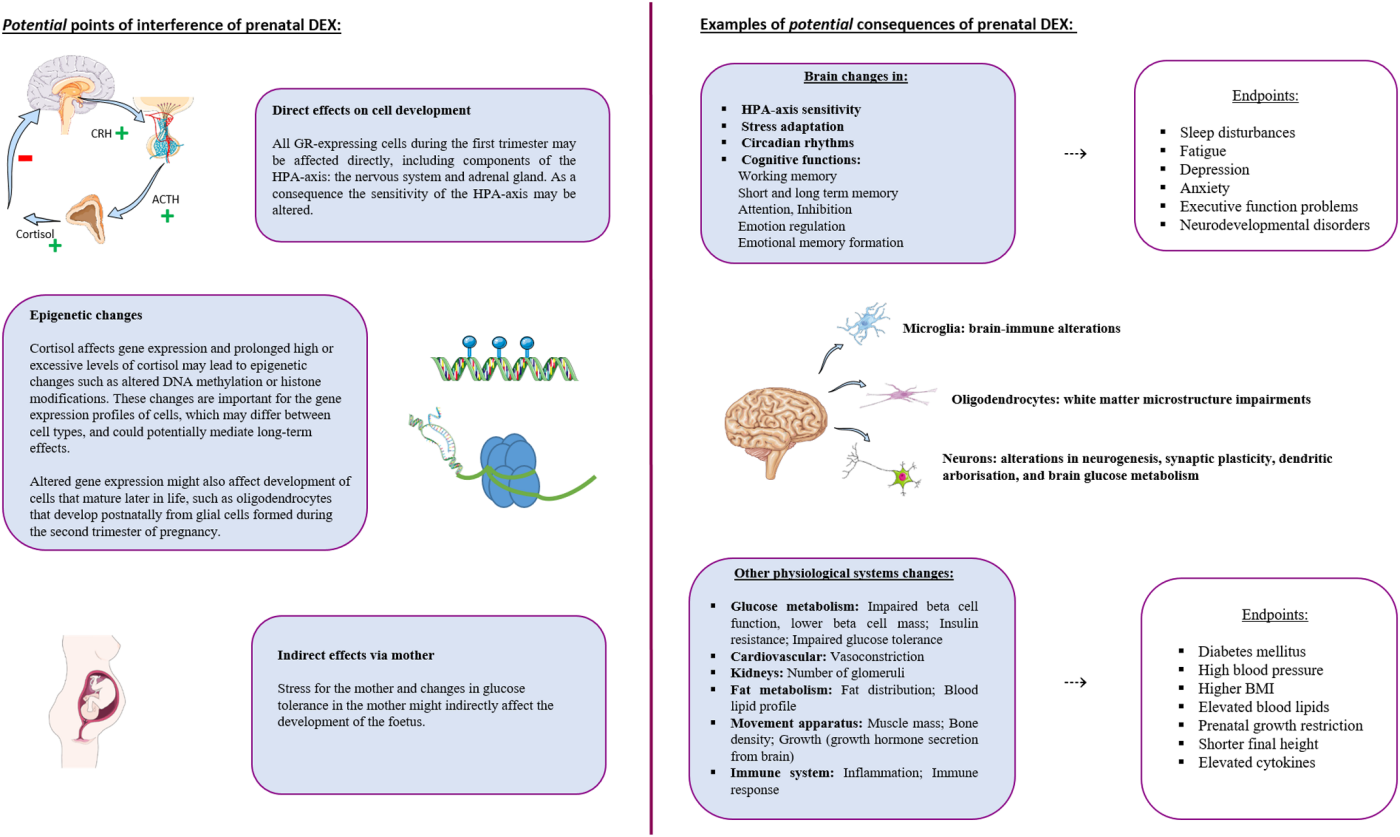
Potential points of interference (left) and potential clinical consequences (right) of prenatal dexamethasone exposure. Parts of the figure were drawn by using pictures from Servier Medical Art. Servier Medical Art by Servier is licensed under a Creative Commons Attribution 3.0 Unported License (https://creativecommons.org/licenses/by/3.0/).

Cortisol is a hormone that is involved in many physiological systems throughout the lifetime. The direct prenatal effects of a high cortisol dose on organ development, its pleiotropic effects and downstream effects as a result of epigenetic programming, as well as indirect effects via the mother, may therefore impact many physiological and psychological functions. In other words, DEX treatment either during the first trimester, or until term, has numerous points of interference that might affect the development of the child. This may result in altered psychological and physiological functioning after birth, during childhood and even into adulthood, with potentially clinically relevant endpoints. We summarized potential points of interference and endpoints in Fig. 2. The current review will focus mainly on brain development as most follow-up studies have focused on cognitive and behavioural outcomes in DEX-treated children. However, we also cover other endpoints that have been assessed in DEX studies in the context of CAH pregnancies.

## Cortisol during prenatal development

DEX treatment needs to be given so early because of the first-trimester development of the adrenal glands and genitals. The adrenal cortex starts developing already prior to GW5, consisting of tissue that produces large amounts of androgens from the ‘fetal zone’, which are crucially involved in forming the feto-placental unit (39, 40). The adrenal cortex develops under the influence of many factors, including its primary regulator fetal pituitary ACTH (40). Fetal pituitary ACTH has been shown to regulate adrenal steroidogenesis from GW12 (41). ACTH is produced already in GW7–8 (42, 43), and both expression of ACTH and nuclear expression of GR are found in GW7 (43, 44). Fetal pituitary ACTH is therefore hypothesized to regulate cortisol biosynthesis during the first trimester (43, 44). Around the same time, external genitalia develop and a fetal cortisol peak occurs between GW7 and 12, which is necessary to suppress the androgens and ensure female sex development in 46,XX fetuses. Although cortisol is needed to suppress adrenal androgens in females, lack of cortisol does not seem to be fatal, as evidenced by the existence of CAH patients with null mutations. Indeed, after the initial peak, cortisol synthesis is suppressed until late gestation, when it is needed for organ maturation (45, 46). Maternal cortisol may pass into the fetal bloodstream only in relatively small amounts during the first trimester, as it is inactivated by HSD11B2, while later in pregnancy, this enzyme becomes less active and more maternal cortisol is allowed to pass (47, 48). Interestingly, expression of the gene coding for GR (*NR3C1*) increases significantly already in GW4, in particular between days 23 and 40 and plateau after day 40 (49). GCs thus play an important role in prenatal development and their levels are tightly regulated and timed (46). Administering high doses of DEX during the first trimester may therefore have widespread effects on all the tissues expressing GR.

## Prenatal brain development

**Figure 3:**
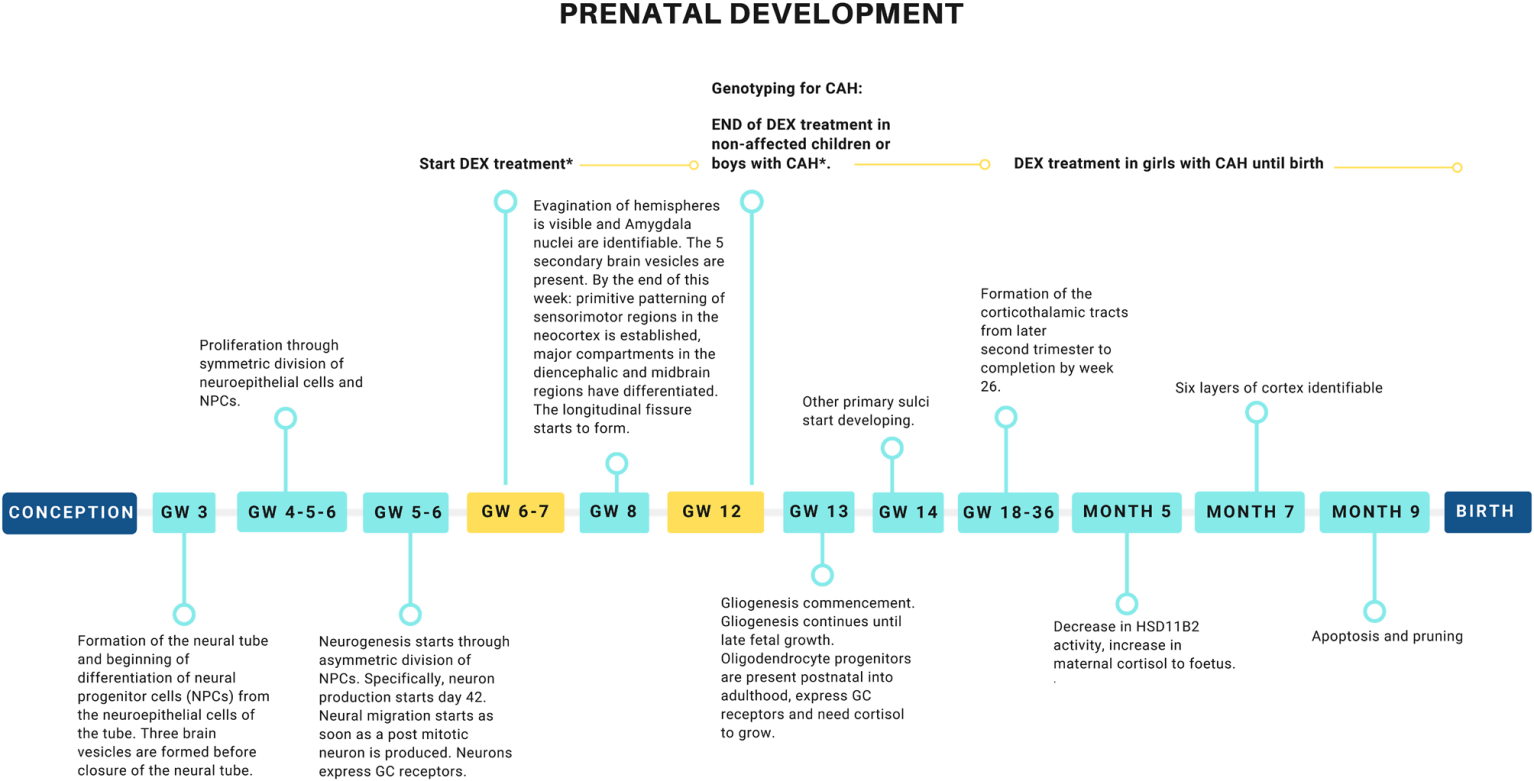
Major steps of prenatal brain development that may be impacted by prenatal dexamethasone exposure. The yellow boxes indicate the start of DEX treatment at GW6–7 and the genotyping for CAH at GW12. Light-blue boxes indicate major events in prenatal brain development. *Although treatment currently is limited to girls because of the possibility of sex-typing prior to treatment, there are large cohorts that have received treatment before the SRY method was available and therefore contain both affected and unaffected boys. GW, gestational week.

Importantly, brain development precedes and coincides with adrenal gland growth. Prenatal brain development and its regulation by GCs have been reviewed extensively elsewhere (50, 51). Figure 3 summarizes the major steps in prenatal brain development. Briefly, during GW3, the process of gastrulation has already resulted in a three-layered embryo including a neural plate, from which the neural tube forms, with the last segment closing on day 27. The three brain vesicles (prosencephalon, mesencephalon, rhombencephalon) form already before the closure of the tube. The neural progenitor cells lying along the neural tube are formed through asymmetric division of neuroepithelial cells, thus creating more neural stem cells that form the ventricular zone during GW4–5. From day 42 (GW6), asymmetric division results in the production of neurons from the subventricular zone. Neurogenesis commences during this period and neurons formed as early as GW6 are known to express GRs and are therefore sensitive to cortisol (49, 52). GW7–12 is marked by neurogenesis and neuronal migration and by GW8, the five secondary brain vesicles are present and the major compartments of the midbrain have differentiated. Interestingly, already at GW8.5, when evagination of the hemispheres becomes visible, the amygdala has formed and all but one nucleus can be identified (53, 54). This region expresses GRs in a high density (55, 56). Formation of the longitudinal fissure also starts in GW8 but develops until GW22. Gliogenesis, that is, the formation of non-neuronal cells, commences a little later, around GW13. However, oligodendrocytes that will form the white matter of the brain have progenitor cells into adulthood and myelination occurs mostly postnatally (57, 58). Importantly, oligodendrocytes express GRs and require the presence of cortisol to develop and might therefore be sensitive to long-term changes in HPA axis function (57, 58).

## Prenatal programming

Considering the above, DEX treatment in the cases of boys and healthy/non-CAH fetuses, started GW7 and lasting until GW12, may be expected to directly affect neurogenesis, neuronal migration and particularly the subcortical structures that form early during gestation. In addition, white matter development may be affected through epigenetic effects either on progenitor cells or other programming effects and may be in particular affected in girls treated until term. Moreover, functional brain networks may be altered as a result of structural changes or other effects on neurotransmitter, hormonal or immune function. DEX treatment may be expected to either directly affect the developing cells or result in changes in physiological systems due to prenatal programming effects.

The Barker hypothesis poses that early life impacts have a cascade of effects on a variety of physiological systems (59). The earlier the impact, the more widespread the effects as the changes may involve epigenetic effects on stem cells. These early life impacts may result in or predispose to adult life disease (60). Moreover, as organs such as the brain aim at finding and maintaining homeostasis, compensatory responses to early life insults may emerge, resulting in a reorganization of the brain networks or adaptive changes in other physiological systems (61, 62). Although such reorganizational changes may not necessarily be harmful, but rather adaptive, or at least compensatory, it is generally thought that early life insults predispose to later disease, in particular in the case of DEX treatment, which entails an exposure to a very high and unnatural amount of GCs. Nonetheless, it is important to consider if any observed changes in the level of the brain appeared as a result of compensation and should therefore always be related to behavioural and cognitive outcomes within long-term follow-up study designs.

## Epigenetic effects

One of the mechanisms through which early life exposure can have long-lasting effects is epigenetic changes such as altering DNA methylation and histone modifications. DEX exposure may alter the expression of genes coding for steroidogenesis as well as GRs and mineralocorticoid receptors and their regulators, which in turn could lead to changes in HPA axis functioning (52, 63). Indeed, offspring of animals treated in late pregnancy with DEX have increased ACTH and cortisol levels, altered HPA axis activity (52, 64) and also displayed changes in stress-related behaviour such as increased anxiety and depression, in particular in females (65). In humans, specifically, neonates born term, who had been treated with betamethasone in the last trimester due to a risk of being born preterm, 505 differentially methylated CpG cites (DMCs) were found in DNA derived from whole blood when compared to non-treated cases. Target genes of these DMCs included genes which had GC response elements in their promotor regions and genes that have a known function in the brain such as *USP48, NTM, MAP6D1, SH3PXD2A, CAMK2N2* (66). This study, therefore, showed that prenatal GC exposure may affect synaptic plasticity and neural organization through epigenetic mechanisms.

Epigenetic effects of synthetic GCs may also be hypothesized to affect placental function. Naturally varying cortisol levels due to maternal stress seem to affect placental function and change methylation of the HSD11B2 gene, which results in increased passage of cortisol into the fetus (67, 68). Hence, DEX administered to mothers, even only during the first trimester, could potentially change placental function through such epigenetic mechanisms, thereby allowing more cortisol to enter later in pregnancy, even when DEX treatment has already been stopped. Epigenetic alteration of the placenta by DEX might therefore be of interest to be investigated.

Interestingly, *in vitro* studies have shown that cortisol administration to hippocampal progenitor cells results in favouring astrocyte- over neuronal cell proliferation during the proliferation stage. However, the progenitors seem to be less affected during the differentiation stage (69, 70, 71, 72). Recently, Cruceanu and colleagues (49) performed a study in which they grew human organoids of neural tissue until gestational day 45 (GW6) when GR expression had stabilized. Treating the organoids with DEX resulted in decreased expression of genes related to neurons, namely *NEUROD6, FOXG1, TBR1, MYT1L* and* NFIA*, and an increase in *PAX6* and *MGARP* normally expressed in neural progenitor cells. Upregulation of genes regulated by GCs, namely *FKBP5, SGK1, TSC22D3* and* ZBTB16,* was observed several days after DEX exposure*.* The effects were specific to neurons (49). These studies show that prenatal alterations in cortisol can have long-lasting effects.

## Models of prenatal excess cortisol exposure in humans

Studies in humans on the effects of other prenatally administered GCs, mainly betamethasone, as well as on naturally varying cortisol levels in cases of maternal stress, have already given insights into the potential effects of high prenatal cortisol exposure on child development. The effects of synthetic GC administration and maternal stress on later neurodevelopmental disorders have been reviewed elsewhere (52, 73, 74). In sum, both prenatal synthetic GCs and naturally varying maternal cortisol levels have been associated with changes in brain structure, function and behaviour in the offspring. However, these studies have mostly considered cortisol exposure during the second and third trimesters.

### Betamethasone exposure during late gestation

Although earlier studies found normal development and no psychological or cognitive problems in children treated antenatally with betamethasone due to a risk of being born preterm (75, 76, 77), more recent studies found cortical thinning of the anterior cingulate cortex associated with affective problems (78), altered glucose metabolism (79) and stress reactivity (80), as well as a change in autonomic nervous system activity, although no differences in salivary cortisol in response to social stress were found (81). Moreover, children exposed to antenatal synthetic GCs have been found to have an increased risk having of any mental or behavioural disorder during the lifetime (81, 82, 83), as well as neurodevelopmental disorders, such as ADHD (81, 82, 83), and have found to have somewhat lower IQ (81). These studies show that synthetic GC exposure has the potential to lead to alterations in brain and behaviour, as well as other endpoints, later in life.

### Maternal stress

Prenatal maternal stress has been linked with long-term changes in the immune system in the offspring, including increases in pro-inflammatory cytokines such as TNF-α (84), as well as higher insulin levels and BMI (85), altered DNA methylation of the GC receptor gene (86), as well as other HPA axis-related genes (87), problems with cognition in children (5.5 years) (88), emotionality in children (89), but also with changes in brain structure and function (90, 91), including amygdala and hippocampal volumes (92) and amygdala connectivity (93). However, studies on maternal stress are confounded by genetic and environmental factors, and the effects are not only due to increased cortisol levels in the mother, although GCs probably do play a significant part in the mediation of prenatal stress-related outcomes in offspring (52).

## Outcomes of prenatal DEX treatment in CAH

Studies on prenatal first-trimester and full-term DEX treatment have evaluated both the effects on the fetus as well as on the mother, assessing many physiological and psychological end points (Fig. 2) (Supplementary Table 1, see section on supplementary materials given at the end of this article).

### Effects of DEX treatment on the mother

Naturally, treatment safety for the mother herself also needs to be considered, both for her own sake as well as for the child, as the developing fetus may also be impacted indirectly via the effects of the high cortisol dose on the mother. Although treatment so far has not been connected with a higher prevalence of gestational diabetes or hypertension in treated mothers, one study found that one-third of women indicated that they would not take the treatment again due to GC-induced side effects (24). Although adverse effects in mothers have not been extensively investigated, the studies that did assess these found that on the short term, common reversible side effects in treated mothers included oedema, severe striae, sleep disturbances and weight gain in particular during the first trimester, with mothers treated full-term tending to report more side effects than those treated only during the first term (24, 36). Sometimes Cushingoid facial features are reported, as well as hypertension, irritability, gastrointestinal intolerance and a hyperglycaemic response to oral glucose administration in a few cases (94). Steroid-induced gestational diabetes could directly affect the child prenatally and result in developmental disorders later in life, which is therefore important to monitor (95).

### Effects of prenatal DEX treatment on the child

Supplementary Table 1 summarizes the outcomes in terms of cognitive functioning, behaviour, MRI and metabolic findings in DEX-treated subjects, including both CAH and non-affected treated individuals. The primary aim of prenatal DEX treatment is reducing virilisation in girls with CAH. However, in addition to reducing virilisation, DEX treatment until term may affect other physiological and psychological functions as well in the affected girls. The primary difficulty in evaluating the effect of prenatal DEX treatment is the small number of participants. This is particularly the case for the girls with CAH, as these make up only one-eighth of study participants in prenatal DEX studies. Most publications contain fewer than ten full-term treated girls, although the French study includes 17 full-term treated girls (31).

#### Outcomes of prenatal DEX treatment in girls and boys with CAH

Generally, the full-term DEX treatment is successful in reducing or completely preventing genital virilisation in girls with CAH. While children with CAH have a greater birth weight and birth length compared to population controls, this is not the case for DEX-treated patients. Although no statistics have been performed (due to the small sample size), DEX-treated CAH cases appear to have smaller birth weight both compared to untreated patients and compared to healthy controls, which indicates growth restriction (24, 96).

In the small cohort of 11 CAH DEX-treated patients (7 females treated until term), no differences in DNA methylation of CD4+ T-cells were identified (97) compared to non-DEX-treated CAH controls. The DEX treatment did not seem to have a visible long-lasting effect on the CAH cases in terms of gene methylation of immune cells (97). That is, for this small cohort with an age range between 5 and 16 years. Recently, Kim and colleagues have found altered methylation in whole blood cells of neonates at risk for preterm birth that had been treated with betamethasone (66). Potentially, methylation changes with age, and it would thus be of interest to test methylation in DEX-treated CAH patients at birth.

Findings regarding the outcome of prenatal DEX treatment in patients with CAH in terms of cognition, behaviour and brain structure and function are mixed and may depend on the age when tested. During childhood, 5- to 12-year-old DEX-treated girls (*n* = 8) from the cohort in the US were found to have slower mental processing compared to untreated girls with CAH, but they did not differ in terms of other cognitive estimates (98). In addition, the Swedish group found lower verbal intellectual ability in 7- to 17-year-old girls (*n* = 6) with CAH that had been treated until term compared to non-treated girls with CAH (96), but no problems with behaviour or mood (99). While one of the first studies (*n* = 2 girls, 3 boys) in the US initially found negative effects of DEX in terms of behaviour (25), this was not repeated in a later study on this cohort (*n* = 31 girls, 17 boys), where children with CAH performed equally well as non-treated patients (100). No internalizing or externalizing problems were found in the initial Swedish cohort (*n* = 4 girls, 5 boys) (101) and not in the Polish cohort either (*n* = 9 girls) (26). However, effects on behaviour may be sex-specific, as in the Swedish cohort, DEX-treated boys (*n* = 5, age 7–17 years) did have more social problems compared to non-treated male patients (99).

In contrast to these studies reporting lower scores on cognition, but no problems in motor and social development, or internalizing symptoms, better performance on visuo-spatial working memory and verbal IQ in full-term treated girls with CAH (*n* = 9) compared to non-treated girls with CAH was found in the Polish cohort (26). However, this study compared the DEX-treated cases to their untreated siblings, who were born as the first case in the family. Given the lack of a neonatal screening programme in Poland at that time, the beneficial effects could potentially also have been the results of earlier diagnosis and earlier treatment start in the sibling having received DEX.

A small group of nine DEX-treated patients (*n* = 4 women, 5 men) were tested at adult age in the PREDEX cohort. DEX-treated patients self-reported more problems with executive function, although the number was too small to perform statistical analyses (102). Similarly, treated CAH women scored lower on most cognitive estimates compared to CAH women that had not been treated (102). The Swedish patients also underwent MRI scanning of the brain. In the sample of DEX-treated CAH cases (*n* = 2 girls, 6 boys), we found differences in the structure of the pericalcarine cortex compared to non-treated CAH cases (103).

Taken together, full-term treatment with DEX seems to affect cognitive functions, but to this end, we do not have evidence of any major impact on behavioural development.

#### Outcomes of first-trimester DEX treatment in non-CAH-affected subjects

The numbers of unaffected subjects treated with DEX are substantially larger and hence easier to perform meaningful statistical analyses. Although height, weight, BMI, heart rate and blood pressure were comparable between DEX-treated participants and population controls (104, 105), we did find differences in terms of beta cell function in the Swedish cohort (105). Indeed, in our child–adult cohort, age 5–26 years, DEX-treated healthy subjects (*n* = 40) had lower HOMA-B index compared to controls, which was only the case for girls (*n* = 18). In the participants that were younger than 16 years, the DEX-treated cases (*n* = 7 girls, 12 boys) had higher plasma glucose levels, while the DEX-treated individuals older than 16 years (*n* = 11 women, 10 men) had higher total plasma cholesterol and higher low-density lipoprotein cholesterol levels (105). These findings are in line with those from a French cohort, in which lower beta-cell mass and reduced insulin secretion were found in first-trimester treated subjects (*n* = 9 women, 7 men) (27), while, BMI, anthropometric characteristics, oral glucose tolerance test and insulin sensitivity during clamp studies were similar in DEX exposed and non-exposed adults. These observations suggest that DEX-treated healthy subjects might have slightly impaired beta cell function, which could increase the risk of developing diabetes or cardiovascular problems, even though no clinically relevant endpoint was studied so far.

Most studies in the first-trimester treated unaffected cases are done on cognitive function and behaviour. In terms of behaviour during childhood, one of the oldest studies dates to 1995, where Trautman and colleagues found that DEX-treated children (age 2–3 years old, *n* = 21 non-CAH) scored higher on shyness and emotionality, but lower on sociability, and had more internalizing (and total) problems compared to non-treated healthy controls, while there were no differences in general development and temperament (25). In a larger follow-up study which included three different age groups (0–15 months (*n* = 36), 15 months–6 years (*n* = 89) and 6–12 years (*n* = 44)), no behavioural differences were observed (100). In the Swedish cohort, despite problems with cognitive functioning in girls (see later), the healthy DEX-treated subjects aged 7–17 years (*n* = 16 girls, 18 boys) were well-adjusted and had no problems with internalizing or externalizing behaviours (parent-reported Child Behaviour Checklist (CBCL)), no difference in social anxiety (Social Phobia and Anxiety Inventory for Children-Parent Report, Social Anxiety Scale for Children-Revised) and not in temperament either (Emotionality-Activity-Sociability-Shyness Temperament survey for children (106). Thus, despite initial reports on internalizing problems in toddlers, most studies seem to find no significant effects in terms of behaviour in DEX-treated healthy subjects.

In the first study on cognition from the PREDEX cohort, which included 17 non-CAH DEX-treated subjects (*n* = 10 girls, 7 boys), we found no differences in parent-reported school performance. However, children reported poorer self-perceived scholastic competence and increased social anxiety. In addition, they had poorer verbal working memory (38). Furthermore, DEX treated (*n* = 17, 10 girls, 7 boys) scored higher on parent-report problems with sociability, although no problems with internalizing or externalizing behaviours were found (CBCL) (101). In the same cohort, DEX-treated boys (*n* = 7) without CAH displayed more neutral gender role behaviour (107).

When assessing cognitive performance in a larger cohort, with the same age range of 7–17 years old, non-CAH DEX-treated girls (*n* = 16), but not boys (*n* = 18), performed worse on several cognitive tasks, namely coding, block design, vocabulary, digit span and span board backward, and they self-reported lower scholastic competence (108). At the same time, the cohort (*n* = 59 non-CAH DEX treated) in the US did not find that mental processing was affected in non-CAH DEX-treated children (98). However, considering the findings of all studies, there may be reason for concern as they indicate problems with cognitive functioning in particular in healthy girls treated with DEX.

Thus far, only the PREDEX study in Sweden has followed treated individuals into adulthood. At adult age, DEX-treated participants (*n* = 12 women, 11 men) did not differ significantly from non-treated healthy controls anymore in terms of cognitive functioning, experienced executive functioning and mood (109). Moreover, an improvement in working memory (digit span) and inhibition (Stroop) was observed from childhood to adulthood, suggesting that the DEX-treated children are able to catch up (109). However, as opposed to the CAH cases, in healthy DEX-treated subjects at adult age (*n* = 12 women, 17 men), we did find altered methylation of many genes in peripheral CD4+ T-cells, most of them associated with immune function and inflammation, but also related to steroidogenesis, and sex-specific effects related to SNPs associated with asthma (110). Interestingly, hypermethylation of *BDNF*, *FKBP5* and *NR3C1* was associated with WAIS subscale performance, namely coding, non-verbal intelligence and inhibition, respectively. However, the differentially methylated genes were associated with performance in a sex-dependent manner, which needs to be further investigated in future studies that have the statistical power to split the groups by sex.

Interestingly, the healthy first-trimester DEX-treated subjects (*n* = 9 women, 10 men) also had enlargement of the bilateral amygdala, larger surface area of the left superior frontal gyrus and alterations in white matter microstructure pointing at reduced integrity of some of the major white matter tracts. In addition, white matter changes, specifically increased radial diffusivity, correlated with hypermethylation of the promotor region of the *FKBP5* gene, which codes for a GR (co)-chaperone (103). However, no relationship between brain structure changes and cognitive performance or behaviour was found (103). Further, no differences in functional activity were found in healthy DEX treated (*n* = 8 women, 10 men) compared to controls, neither during a verbal and visuo-spatial working memory task (111) nor in terms of functional connectivity at rest (112).

The discrepancy in structural and functional findings may be explained by the fact that neurogenesis is already ongoing at the time of DEX exposure and is therefore more likely to be affected directly by DEX. In fact, the amygdala is one of the first structures to develop (53, 54) and may therefore be more sensitive. Potentially, as the brain develops normally for the rest of the duration of pregnancy and onwards, the brain may be able to compensate for the change in structure. This idea is strengthened by the improvement in cognitive functioning in DEX-treated healthy (first trimester treated) participants as they reach adulthood. Of note, a change in structure may also be adaptive, but long-term follow-up studies are needed to determine this. The relationship with *FKBP5* methylation is interesting, considering the recent finding from an organoid study, in which different doses of added DEX were also associated with alterations in this same gene (49).

## Weighing the evidence

The evidence of the effects of DEX treatment for girls with CAH is conflicting, with some studies finding worse cognitive function compared to untreated patients, and others finding better performance. Additional studies with larger sample sizes are needed. If DEX has a negative impact on cognition for the girls, this may be a reason to not opt for DEX treatment. At the same time, the benefit of reduced virilisation, not only physiologically but also in terms of psychological well-being, needs to be weighed against potential issues with cognitive functioning and metabolism. Studies are needed that evaluate whether these potential effects on cognitive functioning translate to problems in daily life, such as school and work performance, and well-being. In other words, the real-life implications of the lower scores in terms of cognition need also to be addressed.

Despite substantial changes in brain structure, DEX-treated healthy (non-CAH) subjects seem to catch up in terms of cognitive functioning from childhood to young adulthood and their brains do not seem to differ in terms of activation at rest or during a task at adolescent and young adult age. Again, additional studies in different age groups and larger cohorts are needed. Whether prenatal DEX delays the maturation of the brain in young children (especially girls) and if that stands for the affected performance during childhood needs to be addressed in additional studies. However, we did find that beta-cell function was impaired, and treated cases may therefore be at a higher risk of diabetes.

## Future research directions

Currently, the dose of DEX administered is high, perhaps as much as three times higher than needed (32). Potentially, a smaller dose would have fewer lasting effects on the structure of the brain or on cognitive function in children, while at the same time replacing the missing hormone and thereby providing for a sufficient reduction in virilisation for the girls with CAH. Future studies may assess the differential impact of varying doses of DEX treatment. For this, international multi-centre studies are required and must include development of early fetal genotyping from maternal blood before initiation of treatment, which would eliminate a large part of the dilemma, although outcomes of girls with CAH would still need to be carefully evaluated. Finally, in Europe, DEX has been administered for decades and many cases, therefore, exist already, both CAH-affected and unaffected that have received this treatment. Efforts should be made to investigate the long-term outcome of these individuals in multinational joint initiatives.

## Conclusions

Taken together, though DEX treatment is effective in reducing virilisation, the safety of the currently used dose is still uncertain and long-term follow-up research is therefore needed, including assessing the effects of using a lower dose.

## Supplementary Material

Supplementary Table 1. Section A: Cognition and behaviour, Section B: Neuro-imaging, Section C: Metabolism and epigenetics in children and adults treated with dexamethasone prenatally. The table also shows some data on the cognitive functions in children and adults with CAH and not treated with DEX prenatally; (m, months; y, years).

## Declaration of interest

The authors have nothing to disclose.

## Funding

This work was supported by the Stockholm County Council (ALF-SLL DNR RS2020-0731 to S.L.), Swedish Research Council (DNR 2021-02440 to S.L.), Region Stockholm (clinical research appointment DNR RS 2019-1140 to S.L.), Stiftelsen Frimurare Barnhuset i Stockholm, Samariten, Jerringfonden, Sällskapet Barnavård, the Deutsche Forschungsgemeinschaft (314061271-TRR 205 and 325768017 to N.R.), the German Ministry of Health (2519FSB503 to N.R.), the Eva Luise und Horst Köhler Stiftung and Else Kröner-Fresenius-Stiftung (2019KollegSE.03 to H.N.).

## Acknowledgement

This manuscript has been written in collaboration with the European Reference Network on rare endocrine conditions (EndoERN), specifically the main thematic groups MTG1 (adrenal) and MTG7 (sex development & maturation). Endo-ERN is funded by the European Union within the framework of the EU4H Program.
