# Supplementary material for: An update on the long-term outcomes of prenatal dexamethasone treatment in congenital adrenal hyperplasia
Source: Endocr Connect. 2023 Mar 15;12(4):e220400. doi: 10.1530/EC-22-0400 (PMC10083667; doi:10.1530/EC-22-0400)
Supplement: Supplementary Table 1. Section A: Cognition and behaviour, Section B: Neuro-imaging, Section C: Metabolism and epigenetics in children and adults treated with dexamethasone prenatally. The table also shows some data on the cognitive functions in children and adults with CAH and not treated with DEX  [file supplementary_table_1.pdf]

Supplementary Table 1. Section A: Cognition and behaviour, Section B: Neuro-imaging, Section C: Metabolism and epigenetics in children and adults treated with dexamethasone prenatally. The table also shows some data on the cognitive functions in children and adults with CAH and not treated with DEX prenatally; (m, months; y, years).

**Section A: Cognition and behaviour**	**Cohort**		**Results – between group differences**			**Reference**
**Country**	***DEX treated***	***Control group (no DEX)***	***Behavioural questionnaires***	***Self-rating scales and interviews***	***Neuropsychological assessment by psychologist***	
**USA**	n = 26<br>21 non-CAH<br>5 CAH (2 girls, 3 boys)	n = 14<br>13 non-CAH<br>1 CAH boy	No differences in general development *(R- DPDQ/MCDI)* and temperament *(ITQ/TTQ/BSQ).*<br><br>DEX-treated had higher scores on shyness (p=0.004) and emotionality (p=0.028), lower on sociability (p=0.04) *(EAS),* and more internalizing (p=0.002) and total problems in 2-3 year old children (p=0.048) *(CBCL).*	-	-	**Trautman et al. 1995**
	Age range total group (n = 40): 6 m–5.5 y; Mean age = 2.5 y (±1.3)					
**USA**	n = 36<br>0–15 m	n = 15<br>0–15 m	No differences in general development (*KIDS/ R-DPDQ*).	-	-	**Meyer-Bahlburg et al. 2004**

	n = 89<br>15 m-6 y	n = 126<br>15 m-6 y	No differences in general development (*CDI/R-DPDQ*).	-	-	
	n = 44<br>6–12 y	n = 162<br>6–12 y	No differences on school performance (*CBCL School scale).*	-	-	
	Total n = 174 (48 CAH, 31 girls, 17 boys)	Total n = 313<br>(195 CAH, 100 girls, 95 boys)				
**Sweden**	n = 26<br>Age range 7–17 y<br>M=10.95 y (2.33)<br>17 non-CAH (10 girls, 7 boys)<br>9 CAH (4 girls, 5 boys)	n = 35<br>Age range 7–17 y<br>M=10.38 y (2.33)<br>Population controls (20 girls, 15 boys)	No difference in school performance (*CBCL School scale, parental rating).*	First trimester DEX treated children without CAH had poorer self-perceived scholastic competence *(SPPC, P=0.003, d=0.82)* and increased social anxiety (*SASC-R General, P=0.025*).	No differences in FSIQ (*WISC-III*), handedness, learning or memory (*NEPSY*).<br><br>First trimester DEX treated children without CAH had poorer verbal working memory *(Digit span WISC-III, P=0.003, d=0.95).*	**Hirvikoski et al. 2007**
**Sweden**	Same cohort as Hirvikoski et al. (2007)		No differences in internalizing or externalizing problems (*CBCL*).<br>No differences on shyness (*SPAI-C-P*).<br><br>DEX treated had higher scores on Sociability (*EAS, P=0.042).*	-	-	**Hirvikoski et al. 2008**

**Sweden**	Same cohort as Hirvikoski et al. (2007)		-	First trimester DEX treated boys without CAH had more neutral behaviours (*KI-GRB*, *p=0.04*).	-	**Hirvikoski et al. 2011**
**USA**	n = 67<br>59 non-CAH<br>First trimester DEX<br><br>8 CAH girls (full-term DEX)<br>8 CAH boys (first trim DEX); Age: 5-12 y<br><br>n = 7<br>1 CAH woman (full-term DEX)<br>6 CAH men (first trim DEX)<br>Age: 11-24 y	n = 73<br>47 non-CAH<br><br>15 CAH girls<br>11 CAH boys<br>Age: 5-12 y<br><br>n =13<br>4 CAH women<br><br>9 CAH men<br><br>Age: 11-24 y	-	-	Slower mental processing in full-term DEX treated girls with CAH *(CAHxDEX interaction effect in K-ABC, Sequental processing, p=0.042 and Mental Processing Composite, p=0.039*).	**Meyer-Bahlburg et al. 2012**

**Poland**	n = 17<br>8 non-CAH girls<br>First trimester DEX<br><br><br>9 CAH girls<br>Age: 6.7-23 y<br>M=13 y<br>Full-term DEX	n = 16<br>No population or sibling control girls without CAH<br><br>16 CAH girls<br>M=16 y	No differences in internalizing or externalizing problems between groups (*CBCL*)	-	In general better cognitive performance in full-term DEX treated girls with CAH compared to not DEX treated girls with CAH. Both groups performed within normal range. (*WAIS-R-PL*, *WISC-R*, *AVLT*, *DCS-VLMT*, *Stroop; IQ verbal p=0.00; IQ performance p=0.03; Stroop p=0.05).*<br><br>Lower verbal working memory performance (Digit span) and visual processing (Block design) in DEX treated girls without CAH (no control group).	**Maryniak et al. 2014**

**Sweden**	n = 34 non-CAH (16 girls)<br>Age range: 7–17 y<br>Girls M = 9.69 (2.50)<br>Boys M = 10.45 (3.04)<br><br>First trimester DEX	n = 67 population controls (36 girls)<br>Age range: 7–17 y<br>Girls M = 10.71 (2.79)<br>Boys M = 10.61 (2.17)	-	DEX treated children score lower on scholastic competence *(SPPC, p=0.001)*	First trimester DEX treated girls without CAH perform worse on *WISC- III Coding (p=0.068, d=0.66), Block design (p=0.021, d=0.81), Vocabulary (p=0.014, d=0.84), Digit span (p=0.001, d=1.00) and WMS-III Span board backward (p= 0.034, d=0.72).*	**Wallensteen et al. 2016**
**Sweden**	Same cohort as Wallensteen et al. (2016).<br><br>First trimester DEX in non-CAH children.	Population controls	DEX treated children without CAH are well adjusted and do not exhibit any internalizing or externalizing problems *(CBCL).* No neg effects on social anxiety *(SPAI-C-P, SASC-R)* or temperament *(EAS)*			**Wallensteen et al. 2018**
**Sweden**	n = 9 CAH DEX<br>4 CAH women (full-term)<br>M = 21.6 y (2.1)<br>5 CAH men (first trim)<br>M = 22.9 y (1.8)<br><br>n = 46 CAH ***no DEX***	n = 46 CAH no DEX<br>26 CAH women<br>M = 25.3 y (4.7)<br>20 CAH men<br>M = 23.1 y (5.1)<br><br>n = 58 population controls	-	Lower scores on self-perceived executive functioning in DEX treated patients with CAH (*B-DEFS-SF*). *Small sample size, statistics not performed. Descriptive.*	Lower performance scores on most measures of cognition in full-term DEX treated women with CAH compared to not DEX treated women with CAH	**Karlsson et al. 2017**

	26 CAH women<br>M = 25.3 y (±4.7)<br>20 CAH men<br>M = 23.1 y (±5.1)<br><br>Age range: 16-33 y	Age range: 16-33 y			*(WAIS-IV Matrices, Vocabulary, Coding, Digit span; WMS-III Span board; WMS-III Word list). Small sample size, statistics not performed. Descriptive.*<br><br>Compared to population controls patients with CAH, not treated with DEX, had impaired working memory *(Digit span, p= 0.024, d=0.46; Span board F, p=0.005, d=0.58; Span board B, p=0.003, d=0.63)* and inhibition *(Stroop, p=0.002, d=0.62).*<br><br>Men with CAH had poorer fluid intelligence/ non-verbal logical reasoning *(WAIS-IV Matrices, p=0.033, d=0.64).*	

					Patients with the null genotype had poorer fluid intelligence/ non-verbal logical reasoning *(WAIS-IV Matrices, p=0.042, d=0.98).*	
**Sweden**	n = 23<br>12 non-CAH women (mean 20.3 y)<br>11 non-CAH men (mean 20.9 y)<br>First trimester DEX<br><br>Age range: 16-33 y	n =58 population controls<br>31 women (mean 20.2 y)<br>27 men (mean 21.3 y)<br><br>Age range: 16-33 y	No differences on behavioral measures – anxiety/depression/autistic traits between first trimester DEX treated adults without CAH and controls *(LSAS, HADS, MADRS, AQ10)*	No differences in self-perceived executive functioning in DEX treated adults without CAH (*B-DEFS-SF*)	First trimester DEX treated adults without CAH perform equal to population controls on cognitive scales *(WAIS-IV Matrices, Vocabulary, Coding, Digit span; WMS-III Span board; Stroop interference test; WMS-III Word list).*<br><br>Improvement in verbal working memory *(Digit span, p=0.04, d=0.58)* and in impulse inhibition *(Stroop interference test, p=0.01, d=1.13)*, but not in general	**Karlsson et al. 2018**

					intelligence over time from childhood to adulthood *(WISC/WAIS-Block design, Matrices, Vocabulary, Coding).*	
**Sweden**	n=11<br>11 CAH DEX<br>6 girls (full-term)<br>5 boys (first trim)<br><br>*n=32*<br>*32 CAH* ***no DEX***<br>*17 girls*<br>*15 boys*	n=32<br>32 CAH no DEX<br>17 girls<br>15 boys<br><br>n=52 population controls<br>27 girls<br>25 boys	-	-	Girls with CAH treated with DEX full-term performed worse on almost all measures compared with non DEX treated girls with CAH.	**Messina et al. 2020a**

	Age range: 7-17 y<br>Mean age ~ 11 y	Age range: 7-17 y<br>Mean age ~ 11 y			Significantly lower verbal intellectual capacity *(WISC-III-Vocabulary, p=0.037, d=1.75).*<br><br>Normal intellectual ability in children with CAH not treated with DEX *(WISC-III Block design, Vocabulary, Coding, Digit span; Span board test; Stroop interference test; NEPSY list learning).*<br><br>Patients with SW CAH, not treated with DEX, performed less well on *Block design (p= 0.058, d=1.25), Span board F (p=0.039, d=1.20) and Span board B (p= 0.056, d=1.03)* than patients with SV CAH, although still within normal range; large effect	

					sizes.	
**Sweden,**<br><br>**Italy**	n=13 CAH DEX<br>8 girls (full-term)<br>5 boys (first trim)<br><br>*n=44 CAH* ***no DEX***<br>*22 girls*<br>*22 boys*<br><br>Age range: 7-17 y<br>Mean age ~ 11 y	n=44 CAH no DEX<br>22 girls<br>22 boys<br><br>n=72 population controls<br>38 girls<br>34 boys<br>Age range:7-17 y<br>Mean age ~ 11 y	More social problems in DEX treated boys with CAH compared to not DEX treated boys with CAH *(CBCL-social problems, p=0.003).* No other behavioral or mood problems in DEX treated children with CAH *(CBCL internalizing and externalizing problems; CBCL total problem score; SPAI-C-P; EAS; SASC-R).*<br><br>Girls with CAH (non-DEX) were rated by their parents to have more social problems *(CBCL-social problems, p= 0.067).*	No differences in self-perceived scholastic competence *(SPPC).*	-	**Messina et al. 2020b**
**Section B: Neuro-imaging**	***DEX treated***	***Control group (no DEX)***	***Neuro-imaging findings***	***Associations neuro-imaging findings and other variables***	***Cognition & Behaviour results within Imaging studies***	

**Sweden**	n=8 CAH DEX<br>2 females (full-term)<br>6 males (first trim)<br><br>Age range: 19-26 y<br>Mean age: 22.3 y	n= 37 CAH no Dex<br>21 females<br>16 males<br><br>n=43 population controls<br>26 females<br>17 males<br><br>Age range: 16-33 y<br>Mean age: 23.2 y	DEX-treated patients had reduced surface area of the bilateral pericalcarine cortex, reduced volume of the left pericalcarine and right superior parietal cortex compared to untreated CAH patients.<br>No differences in white matter.			**Van't Westeinde et al., 2019**
**Sweden**	n= 19 Healthy DEX<br>9 females (first trim)<br>10 males (first trim)<br><br>Age range: 16-26 y<br>Mean age: 20.7 y	N=43 Healthy No DEX<br><br>Age range: 16-25 y<br>Mean age: 20.3 y	DEX-treated had bilateral larger amygdalae, increased surface area and volume of the left superior frontal gyrus.<br><br>DEX-treated had increased radial, mean and axial diffusivity in the major white matter tracts, in particular the superior longitudinal fasciculi and corticospinal tracts, pointing at impairments in white matter.<br><br>Sex specific effects:<br>DEX treated men had	Within the DEX-group, increased radial diffusivity of white matter correlated with increased methylation of the promotor region of the *FKBP5* gene.<br><br>No differential relationship between cognition and brain structure in DEX vs untreated.	No differences in anxiety or cognitive functioning between DEX and untreated.	**Van't Westeinde et al. 2020a**

			increased AD, while DEX-treated women had reduced FA in major white matter tracts.			
**Sweden**	n=18 Healthy DEX<br>8 females<br>10 males<br><br>Age range: 16-26 y<br>Mean age: 20.8 y	n=40 Healthy NO-DEX<br>24 females<br>16 males<br><br>Age range: 16-25 y<br>Mean age: 20.5 y	No differences in brain activity during verbal or visuo-spatial working memory.		DEX-treated responded faster on the verbal working memory task	**Van't Westeinde et al. 2020b**
**Sweden**	N=18 Healthy DEX<br>8 females<br>10 males<br><br>Age range: 16-26 y	N=38 Healthy NO-DEX<br>24 females<br>14 males<br><br>Age range: 16-26? y	No differences in resting-state activity of the brain			**Messina et al. 2022**
**Section C: Metabolism and epigenetics**						
**Sweden**	N=29 Healthy DEX<br>12 females<br>17 males<br><br><br>Mean age: 16.4 y<br>Age SD: 5.9 y	N= 37 Healthy NO-DEX<br>18 females<br>19 males<br><br>Mean age:17 y<br>Age SD: 6.1 y	9672 differentially methylated probes were associated with DEX treatment in CD4+ T cells.<br><br>7393 probes were differentially methylated depending on sex.<br><br>Functional enrichment mostly in immune-related genes.	Methylation in the *BDNF*, *FKBP5* and *NR3C1* genes were associated with performance on subscales of the WAIS IQ test.		**Karlsson et al., 2019**

			DMPs near SNPs were associated with inflammatory bowel disease.<br><br>DEX x SEX were enriched near SNPs associated with asthma.<br><br>DPMs in genes involved in regulation and maintenance of methylation and steroidogenesis.			
**Sweden**	n=40 Healthy DEX<br>18 females<br>22 males<br><br>Age range: 5.1-26.4 y<br>Mean age: 16.3 y	n= 75 Healthy NO-DEX<br>35 females<br>40 males<br><br>Age range: 4.5-26.6 y<br>Mean age: 16.3 y	DEX-treated had no difference in blood count, renal function or serum lipid profiles.<br><br>DEX treated had lower HOMA-B index only in girls.<br><br>DEX-treated < 16 years had higher leukocyte levels, lower HOMA-B, higher plasma glucose and higher potassium levels.<br><br>DEX-treated > 16 years had higher total plasma cholesterol and higher low-density lipoprotein cholesterol levels			**Wallensteen et al., 2020**

**France**	n=16 Healthy DEX exposed<br>9 females<br>7 males<br><br>Mean age: 24.6 y<br>Age SD: 3.5	n=16 Healthy NO-DEX<br>9 females<br>7 males<br><br>Mean age: 24.3 y<br>Age SD: 3.4 y	DEX-treated did not differ in terms of insulinogenic index during an oral glucose tolerance test and not in insulin sensitivity during clamp either.<br><br>In DEX-treated insulin secretion decreased by 17% during graded IV glucose infusion, and with 22% after arginine administration. Glucagon secretion after aginine increased.			**Riveline et al., 2020**
**Sweden**	n=33 Healthy DEX<br>16 females<br>17 males<br><br>Age range: 5.1-26.3<br>Mean age: 15.7<br><br>n=19 < 18 years	n=54 Healthy NO-DEX<br>28 females<br><br>Age range: 5.5-25.3<br>Mean age: --<br><br>n=27 < 18 years	Age, height, weight and body mass index did not differ between groups.<br><br>Heart rate, 24-hour BP, pulse pressure, and nighttime dipping did not differ between groups.			**Karlsson et al., 2022**

Note: R-DPDQ = Revised Denver Prescreening Developmental Questionnaire; MCDI = Minnesota Child Development Inventory; CBCL = Child Behaviour Checklist; ITQ =Infant Temperament Questionnaire; TTQ = Toddler Temperament Questionnaire; BSQ = Behavioral Style Questionnaire; EAS = EAS Temperament Survey for Children; KIDS = Kent Infant Development Scale; CDI = Child Development Inventory; WISC-III = Wechsler Intelligence Scales for Children; NEPSY = Developmental Neuropsychological Assessment; SPPC = Self-Perception Profile for Children; SASC-R = The Social Anxiety Scale for Children–Revised; SPAI-C-P = Social Phobia and Anxiety Inventory for Children – Parent Report; KI-GRB = The Karolinska Inventory of Gender Role Behaviour. K-ABC = Kaufman Assessment Battery for Children; WAIS = Wechsler Adult Intelligence Scale; WMS = Wechsler Memory Scale; B-DEFS-SF

= Barkley Deficit in Executive Functioning Scale – Short Form; LSAS-SR = Liebowitz Social Anxiety Scale-subclinical social anxiety; HADS = Hospital Anxiety and Depression scale; B-DEFS-SF short version = Barkley Deficit in Executive Functioning Scale; MADRS = Montgomery Åsberg Depression Rating Scale; AQ10 = Autism Spectrum Quotient.
